# Successful transition from long-term peritoneal dialysis to intermittent hemodialysis in a patient with Fontan circulation

**DOI:** 10.1007/s13730-026-01091-9

**Published:** 2026-02-03

**Authors:** Ryo Nakatani, Yoko Shirai, Gen Harada, Takeshi Shinkawa, Osamu Segawa, Akinori Masuda, Norio Hanafusa, Kenichiro Miura

**Affiliations:** 1 Department of Pediatric Nephrology, Tokyo Women’s Medical University, 8-1, Kawada-cho, Shinjuku-ku, Tokyo, Japan; 2 Department of Pediatric Cardiology and Adult Congenital Cardiology, Tokyo Women’s Medical University, Tokyo, Japan; 3 Department of Cardiovascular Surgery, Tokyo Women’s Medical University, Tokyo, Japan; 4 Department of Pediatric Surgery, Tokyo Women’s Medical University, Tokyo, Japan; 5 Dialysis Center, Dokkyo Medical University Koshigaya Hospital, Saitama, Japan; 6 Department of Blood Purification, Tokyo Women’s Medical University, Tokyo, Japan

**Keywords:** Fontan circulation, Encapsulating peritoneal sclerosis, Hemodialysis, Peritoneal dialysis

## Abstract

The Fontan procedure is a palliative surgical technique used for complex congenital heart disease resulting in a functional single ventricle. Patients with Fontan circulation often exhibit elevated central venous pressure (CVP) and reduced cardiac output, which make intermittent hemodialysis (IHD) challenging. Between dialysis sessions, increased preload due to fluid accumulation may precipitate congestive heart failure, whereas excessive ultrafiltration can reduce preload and consequently compromise cardiac output. We report the case of a 23-year-old man with a single ventricle of left ventricular morphology who underwent a Fontan procedure at 2 years of age. He developed kidney failure secondary to bilateral hypoplastic kidneys and began peritoneal dialysis (PD) at 12 years of age. PD was continued for 9 years because IHD and kidney transplantation were contraindicated due to cardiac dysfunction with arrhythmia. He presented with a refractory exit-site infection, for which laparoscopic surgery revealed early-stage encapsulating peritoneal sclerosis. Cardiac catheterization revealed a CVP of 11–12 mmHg, and ventricular function was preserved. Based on these findings, transition to IHD was considered feasible. Permanent pacemaker implantation and insertion of a tunneled hemodialysis catheter were performed concurrently. IHD was initiated at a blood flow rate of 150 mL/min, which was well tolerated, with no episodes of hemodynamic instability observed. The patient has since been maintained on outpatient IHD for 9 months without major complications. This case demonstrates that IHD can be safely performed in selected patients with Fontan circulation, provided that careful preoperative hemodynamic assessment is undertaken to confirm adequate cardiovascular stability.

## Introduction

The Fontan procedure is a palliative surgical technique performed in patients with complex congenital heart disease resulting in a functional single ventricle [1]. This circulation relies on passive pulmonary blood flow driven by elevated central venous pressure (CVP). Consequently, patients frequently develop long-term complications due to chronically elevated CVP and reduced cardiac output, including liver congestion, arrhythmias, and exercise intolerance [2].

Fontan circulation is characterized by fragility, as the systemic circulation depends on pressure-driven, non-pulsatile pulmonary blood flow; therefore, even minor fluctuations in intravascular volume can markedly affect systemic perfusion [3]. Therefore, in patients with Fontan circulation undergoing intermittent hemodialysis (IHD), there is a concern that an increase in preload between hemodialysis (HD) sessions may precipitate congestive heart failure, whereas excessive ultrafiltration may reduce preload and compromise cardiac output [4]. Furthermore, elevated CVP and impaired ventricular compliance further limit the cardiovascular reserve, leaving only a narrow margin in which IHD can be safely performed [5].

Here, we report a patient with Fontan circulation who was successfully transitioned from long-term peritoneal dialysis (PD) to IHD, with previously reported laparoscopic findings of early-stage encapsulating peritoneal sclerosis (EPS) [6].

## Case report

A 23-year-old man with a single ventricle of left ventricular morphology underwent a Fontan procedure with an extracardiac total cavo-pulmonary connection at 2 years of age. He subsequently developed kidney failure secondary to bilateral hypoplastic kidneys and began PD at 12 years of age. PD was continued for 9 years because he was considered unsuitable for HD or kidney transplantation (KT) owing to impaired cardiac function, with an ejection fraction of 43%, and the presence of concomitant complete atrioventricular block. He remained on automated PD with a daytime dwell, maintaining stable ultrafiltration without any episodes of failure, and his daily ultrafiltration volume was approximately 1000 mL. At 21 years of age, during treatment for a refractory exit-site infection, laparoscopic examination revealed early-stage EPS [6]. Subsequently, the diagnosis of early-stage EPS necessitated withdrawal from PD, prompting reconsideration of IHD as an alternative kidney replacement therapy. During the 9-year PD course, no clinical signs suggestive of EPS, such as bowel obstruction or ultrafiltration failure, were observed. The results of the previous annual peritoneal equilibration tests, performed according to a standard protocol using 2.0 L of 2.5% dextrose solution, had been classified as low-average for the preceding 4 years. However, at the time of this evaluation, a 4 h dialysate-to-plasma creatinine ratio of 0.69, consistent with a high-average transporter profile. Oral prednisolone was initiated for early-stage EPS, and transition to IHD was considered.

On examination, his height was 153.3 cm and body weight was 37.7 kg. Vital signs showed a blood pressure of 123/67 mmHg and a pulse rate of 51 beats per minute. Blood tests revealed a blood urea nitrogen of 81.0 mg/dL, potassium 4.1 mEq/L, calcium 8.9 mg/dL, phosphate 5.1 mg/dL, and a brain natriuretic peptide (BNP) level of 25.0 pg/mL. Chest radiography, demonstrating findings typical of patients with Fontan circulation, showed a cardiothoracic ratio of 41.3% with sharp costophrenic angles, a mildly prominent right first cardiac contour, and slight concavity of the right second cardiac border. Electrocardiography revealed complete atrioventricular block. After consultation with pediatric cardiologists and cardiovascular surgeons, cardiac catheterization demonstrated a mildly elevated CVP (11–12 mmHg; normal range, 2–6 mmHg) and preserved ventricular function with a ventricular ejection fraction of 54%, representing an improvement compared with the value of 43% recorded 9 years earlier. Echocardiography also showed preserved ventricular contractility (fractional shortening 0.41) without pericardial effusion. These findings suggested that IHD could be introduced safely, after completion of atrioventricular block treatment.

The patient also had autism spectrum disorder, attention-deficit/hyperactivity disorder, and moderate intellectual disability (intelligence quotient of 44). Because of narrow veins and difficulty tolerating repeated needle puncture, creation of an arteriovenous fistula (AVF) was considered impractical. Therefore, a tunneled HD catheter was selected as long-term vascular access.

**Fig. 1:**
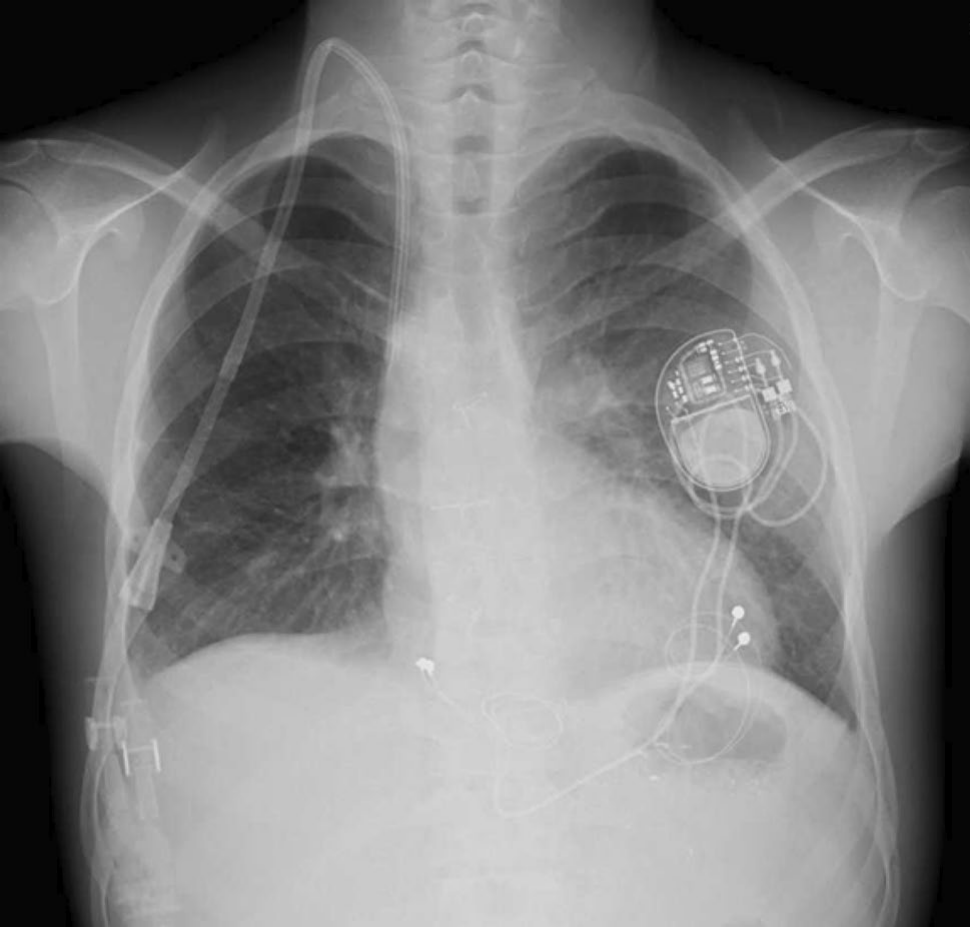
Chest X-ray after insertion of a tunneled hemodialysis catheter. The tip of the tunneled hemodialysis catheter was presumed to be located in the extracardiac Fontan conduit. A permanent pacemaker was implanted at the same time

Permanent pacemaker implantation for complete atrioventricular block and insertion of a 13-French diameter tunneled HD catheter via the right internal jugular vein were performed (Fig. 1). IHD was initiated on the following day, and maintenance HD was performed with a blood flow rate of 150 mL/min. The interdialytic weight gain over a one-day interval was approximately 1 kg, corresponding to about 2.5% of his body weight, with a maximum ultrafiltration volume of 2.4 L. His blood pressure remained around 130–140/60–70 mmHg during HD sessions, without hemodynamic instability. IHD has since been continued for 9 months without major complications. Dry weight has been adjusted according to edema, blood pressure, and BNP levels, which have been maintained below 20–30 pg/mL. In addition, peritoneal lavage has been continued every other day, draining approximately 300 mL of ascitic fluid each time. He will be assessed in the near future regarding the feasibility of KT.

## Discussion

To our knowledge, this is among the few reported cases describing successful introduction of IHD in a patient with Fontan circulation. Patients with Fontan circulation pose unique challenges when kidney replacement therapy is required as elevated CVP, reduced cardiac output, and impaired venous return often limit tolerance to IHD. Previous reports have described the use of continuous hemodiafiltration in patients with Fontan circulation with markedly elevated CVP (around 30 mmHg), but outcomes were poor due to hemodynamic intolerance and unfavorable prognosis [7]. Although some patients with Fontan circulation have undergone continuous hemodiafiltration successfully [7, 8], the hemodynamic thresholds—such as CVP and ejection fraction—that permit safe initiation of IHD have not been clearly defined. In the present case, cardiac catheterization revealed a mildly elevated CVP of 11–12 mmHg with preserved ventricular function. After multidisciplinary discussion with pediatric cardiologists, the patient was considered able to tolerate initiation of IHD. IHD was subsequently introduced without cardiovascular instability, and the patient has remained stable for 9 months. This suggests that, when cardiac function is preserved and CVP elevation is mild, IHD can be safely performed in selected patients with Fontan circulation.

Another important clinical consideration was vascular access. In patients with Fontan circulation, AVF or arteriovenous graft (AVG) may further increase preload and venous pressure, posing hemodynamic risk; to our knowledge, IHD via such access has not been reported in this population. Therefore, when contemplating AVF or AVG creation, it is prudent to evaluate whether patients can tolerate the resultant increase in preload. In our patient, repeated needle puncture was considered difficult due to moderate intellectual disability; therefore, neither an AVF nor an AVG was created. Consequently, a tunneled HD catheter was selected as long-term access and enabled stable IHD. Although maintenance IHD has been stable to date, long-term use of a tunneled catheter carries risks of catheter-related bloodstream infection and thrombosis [9], thus requiring careful surveillance.

Furthermore, the patient developed early-stage EPS after 9 years of PD. Prednisolone was administered in an attempt to prevent disease progression [10]. Peritoneal lavage was also continued after HD initiation because it has been reported to reduce the incidence of EPS following PD withdrawal [10]. Although no consensus exists regarding the optimal duration of peritoneal lavage [10, 11], it will be continued until ascitic fluid drainage decreases. This highlights the importance of individualized management strategies when transitioning from long-term PD to HD in patients with Fontan circulation.

KT may also represent a therapeutic option for patients with Fontan circulation. KT may be considered in selected patients with Fontan circulation when CVP can be adequately controlled and sufficient systemic venous return maintained to ensure appropriate graft perfusion. A recent case report demonstrated that adequate systemic blood pressure can be achieved under fluid loading during cardiac catheterization, thereby ensuring sufficient graft perfusion and enabling successful KT [12]. Accordingly, this patient is scheduled to undergo further hemodynamic assessment with fluid challenge to determine the feasibility of KT in the future.

In conclusion, this case demonstrates that IHD can be safely performed in selected patients with Fontan circulation, provided that careful preoperative hemodynamic assessment is undertaken to confirm adequate cardiovascular stability.

## Data Availability

Data sharing is not applicable to this article because no datasets were generated or analyzed during the current study.
